# Substandard and falsified medicines in Africa: healthcare systems challenges, supply chain issues, regulatory challenges and strategies to increase access to quality medicines

**DOI:** 10.3389/fphar.2025.1708784

**Published:** 2025-11-18

**Authors:** Billy Chabalenge, Tarsem Sahota, Irina Ermolina, Sangeeta Tanna

**Affiliations:** Leicester School of Pharmacy, De Montfort University, Leicester, United Kingdom

**Keywords:** Africa, healthcare systems, falsified medicines, regulatory challenges, substandard medicines, quality medicines and medicines access

## Abstract

Africa is a unique continent due to high disease burden for both communicable and non-communicable diseases, attracting large-scale distribution of medicines to tackle public health issues. This poses several challenges for the healthcare systems, particularly concerning the circulation of substandard and falsified medicines. The widespread of substandard and falsified medicines causes adverse health effects on patients and contributes to millions of deaths annually. This review explores how health systems in Africa, supply chain issues and regulatory environment affect the circulation of substandard and falsified medicines. A narrative review was conducted drawing information from academic publication, official reports and other grey literature between January 2014 and October 2025 focusing on substandard and falsified medicines, health systems, supply chain and regulatory issues in Africa. Health system challenges in Africa include uneven geographical distribution of health facilities, medicines stockouts, increased costs of medicines purchased from private healthcare facilities and inadequate health financing. These factors increase out-of-pocket expenditure on patients and drive the poor majority of Africans to source cheaper medicines from unregulated markets, thereby increasing the likelihood of consuming substandard and falsified medicines. Supply chain issues include a lack of industrialisation to meet the medicine demand, a lack of support to local logisticians, poor forecasting, and over-dependence on imports, complicating the supply of quality medicines. The complexity of the supply chain system creates numerous opportunities to disrupt the supply of quality and safe medicines, making it easy for substandard and falsified medicines to reach the patient. Medicines regulation in Africa also suffers several challenges including limited well-functioning regulatory systems, inadequate staffing levels and a lack of analytical technologies for fast screening of substandard and falsified medicines. The review further provides recommendations and priority areas that should be considered to strengthen health systems, supply chains and regulation of medicines in Africa to reduce the prevalence of substandard and falsified on the continent.

## 1 Introduction

Substandard medicines–also called ‘out of specification’, are “authorised medicines that fail to meet either their quality standards or their specifications, or both” whereas falsified medicines are medicines that “deliberately/fraudulently misrepresent their identity, composition, or source” (World Health Org anisation, 2017). Substandard medicines are a consequence of poor quality-control and manufacturing practices, whereas falsified medicines are produced and labelled with the deliberate attempt to defraud consumers. In reality, both types of poor-quality medicines claim to be something they are not (Lawson et al., 2018). Substandard and falsified (SF) medicines pose a global threat to public health and hinder the achievement of universal health coverage and sustainable development goals (Salami et al., 2023; Stevens et al., 2023). The global prevalence of SF medicines is estimated to be between 10.5% and 25% of the medicines, with higher-income countries reporting only about 1% of the medicines to be substandard or falsified (World Health Organisation, 2017a; Melia et al., 2024). African nations, where healthcare systems and regulatory capacities are often inadequate, are affected more than other continents, with the prevalence of SF medicines over the last 2 decades estimated to be as high as up to 70% of all medicines supplied in some regions (Aminu et al., 2017). A study by Hajjou and co-workers showed that 10.4% of African medicines were substandard between 2003 and 2013 (Hajjou et al., 2015). An increase in the prevalence of SF medicines to about 42% in Africa was also observed by WHO between 2013–2017 through the Global Surveillance and Monitoring System (World Health Organisation, 2017a). Another study analyzed various reports of SF antimicrobials between 2017 and 2023 and found that 22.6% of antimicrobials in selected East African nations are either substandard or falsified medicines (Tegegne et al., 2024). A systematic review analyzing 27 studies across the African continent found the prevalence of SF medicines to be 22.6% (Asrade Mekonnen et al., 2024). More recently, a study conducted to assess the quality of antidiabetic medicines in 13 sub-Saharan African countries revealed that 28% of the 1,673 tested samples failed the assay test (Antignac et al., 2025). In Africa, antibiotics, antimalarial, anthelmintic, antihypertensives and antiprotozoal are the most reported therapeutic groups to be of such poor quality and this is attributed to the high demand for these medicines due to disease prevalence in most regions of Africa (Tegegne et al., 2024; Asrade Mekonnen et al., 2024).

SF medicines have devastating consequences on patients because they often contain insufficient or incorrect amounts of the active pharmaceutical ingredient(s) (APIs), contain no APIs, are degraded medicines or contain other toxic substances or impurities. These consequences include increased morbidity, antimicrobial resistance, adverse drug reactions, treatment failure, non-intentional medication non-adherence, a decline in trust in the healthcare system, health complications, and even death (World Health Organisation, 2017b; Tanna and Lawson, 2016). For example, approximately 169,000 children die from pneumonia annually, with an additional 158,000 succumbing to malaria in Africa due to the use of SF medicines (World Health Organisation, 2017b). In 2022, over 66 deaths were recorded associated with substandard paediatric cough mixtures and related products contaminated with diethylene glycol (Thiagarajan, 2022). SF medicines also exert a direct economic and socio-economic impact on individual patients, health systems resources, pharmaceutical industries, the environment, and Africa governments (World Health Organisation, 2017b; OECD, 2020). At the patient level, treatment failures, drug toxicity, and adverse events caused by SF medicines result in heightened out-of-pocket expenses and even death. This is because of increased medical investigations, prolonged hospital stays, and additional travel costs for those seeking quality healthcare outside a system in which they have lost confidence (World Health Organisation, 2017b; Popoola et al., 2022). Additionally, the extended sickness results in decreased productivity, affecting the patient, their caregivers and the broader economy, potentially reinforcing the cycle of poverty as patient income is reduced (Salami et al., 2023). On healthcare system resources, SF medicine causes increased burden and spending as a result of transmission of resistant infections and increased hospitalisations. Governments lose hundreds of millions in taxes from the smuggling of SF medicines in black markets (Karungamye, 2023). In East African countries alone, the burden was estimated to cause a loss of around US$500 million in unremitted taxes (Aminu et al., 2017; World Health Organisation, 2017b). Licensed pharmaceutical manufacturers are not spared by the impact of SF medicines as they incur losses in their sales, their market shares and their business opportunities are also diminished (Karungamye, 2023). When prescribers and patients continuously observe treatment failure from the use of a brand medicine which may have been falsified, they are less likely to prescribe or consume the same medicine in future leading to loss of market for the manufacturers.

In the last 20 years there appear to be no reduction in the prevalence of SF medicines in Africa despite several actions taken. Therefore, access to quality-assured medicines in Africa is paramount in improving health outcomes. In Africa, health systems structures, medicines supply chains and regulatory environments all play a critical role in patients accessing quality and safe medicines. However, the prevalence of SF medicines in Africa affects the performance of the health systems. Understanding how these health systems impact access to quality and safe medicines for patients is essential in providing strategies for preventing and detecting SF medicines in Africa. While previous reviews have primarily described the prevalence and general causes of SF medicines in Africa, this paper aims to evaluate how the health systems in Africa, supply chain issues and regulatory environment affect the circulation of SF medicines in the continent. Further, the review provides recommendations that should be considered to strengthen health systems, supply chains and regulation of medicines in Africa and improve access to quality medicines in order to reduce the growing threat of SF medicines on the continent.

## 2 Methodology

To evaluate how health systems, supply chain issues and regulatory environment affect the circulation of SF medicines in Africa, the method adopted by this study is similar to that used by other scholars in this field such as Adebisi et al. (2022).

This review started with searching published literature, reports and gray literature in both international and Africa journal articles for relevant papers on the subject that were published from January 2014 to October 2025. Several databases were used during searching, including Pub Med, Google scholar, MEDLINE, and EMBASE. A manual search in google was also conducted to collate relevant regional health authorities documents, policy briefs, news and reports. Further, reports from International Organisations such as the World Health Organisation, United Nations Conference on Trade and Development, and the Institute of Economic Justice were collated. Relevant articles were extracted using keywords such as “Substandard,” “falsified,” “medicine access,” and “challenges” combined with contextual keywords such as “Africa,” “health systems,” “medicines quality,” “supply chains,” and “regulatory.” To ensure all relevant articles were collected, the Boolean operators AND and OR were applied.

The search was limited to studies that were published in English language from January 2014 to October 2025, as this period saw an increase in publications on SF medicines in Africa. Additionally, this period was focused on to ensure the findings reflect the current challenges and impact of healthcare systems, supply chain and regulatory environment in Africa on SF medicines and access to quality medicines. Studies focusing solely on non-African context and without clear reference to medicine quality were excluded. The information extracted was organised and discussed under three thematic areas; health systems, supply chain, and regulatory issues.

## 3 Healthcare systems in africa and their impact on the circulation of SF medicines

Patients in Africa access medicines and other healthcare services from public health institutions largely owned by the governments and private institutions which are privately owned or managed. However, the geographic distribution of the public and private health sectors varies from country to country and have different impact on the access to quality medicines and, ultimately the circulation of SF medicines.

### 3.1 The structure of the public healthcare system in africa

**FIGURE 1:**
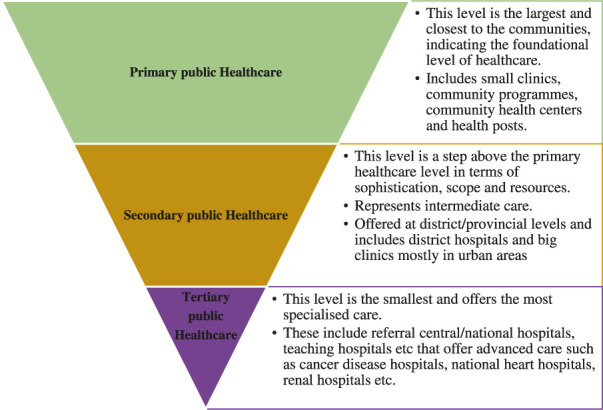
Hierarchy of public healthcare system structures in African countries. The referral system moves from primary healthcare to tertiary healthcare facilities.

The architecture of most of the public health institutions in Africa is structured in different hierarchical levels (Figure 1) through established referral systems from the lowest facilities to highly specialised facilities (Falchetta et al., 2020; Toroitich et al., 2022). However, the governance functionality of these public health institutions differs from country to country. For instance, in Tanzania, decentralised healthcare systems give much power to local political councils and communities, whilst in countries including Kenya, Zambia, Mozambique, and Malawi, the public healthcare systems have devolved governance structures that still depend on the central government (Falchetta et al., 2020; Toroitich et al., 2022; Kigume and Maluka, 2019). In these systems, the central ministry of health are the custodian of health policy, planning, and financing of these levels of healthcare while implementation of health programmes and healthcare services remain the responsibility of each level of healthcare.

### 3.2 The structure of the private healthcare system in africa

**FIGURE 2:**
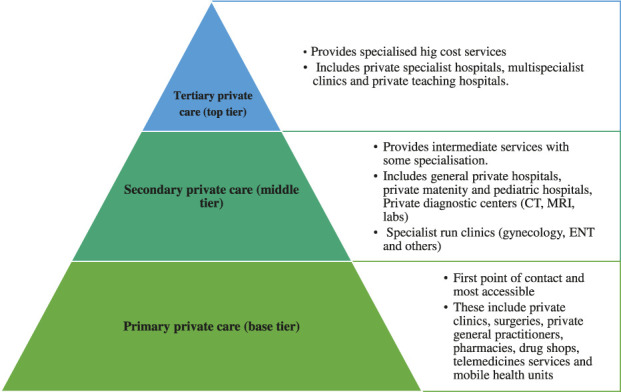
Functional structure of private healthcare institutions in Africa. Investment is often by Self-funding, private investors, non-governmental organisations, and Faith based organisations.

The private sector in most African countries is heterogeneous (Figure 2), comprising various players including not-for-profit non-government healthcare providers, for-profit private healthcare providers and, in some cases, a mix of public-private partnerships. The services offered follows similar functional hierarchy to public healthcare systems but mainly shaped based on investment, accessibility and market demand as opposed to strict national policies. Some key differences between the public healthcare structure and private healthcare structures are highlighted in Table 1.

**TABLE 1:** Key differences between the public and private healthcare systems in Africa.

Key feature	Public healthcare	Private healthcare
Governance	Structured and overseen by national, provincial and district authorities	Varies widely from one institution to the other
Organisation/structure	Well defined primary, secondary and tertiary levels based on national policies	Fragmented but loosely hierarchical driven by local demand, service profitability and investor interests
Fund sources	Primarily government budgets, some public health insurance, donor funding, and international aid	Out-of-pocket payments, private insurance, and investor capital. Some non-governmental organisations (NGOs) and faith based organisations (FBOs) receive donor funding
Access and equity	Aimed at national coverage to achieve universal health coverage. Some services are usually subsidized or free especially at primary level	Access restricted by income levels, insurance coverage and geographical location Equity is a challenge unless subsided usually by NGOs and FBOs

The private healthcare systems have significant involvement in health service delivery in African countries as they often exist and operate in parallel to public health institutions (Fanelli et al., 2020). For instance, 50% of all healthcare services in sub-Saharan Africa are delivered by the private sector (Awor and Kinengyere, 2023). However, access to private healthcare services varies across African regions, with only 35% of patients going to for-profit private facilities such as community pharmacies, while 17% go to not-for-profit and informal providers (Montagu and Chakraborty, 2021). This is because private healthcare institutions are still limited with the majority of the population failing to afford the services. As a result, the majority are located in urban areas and only a few located in rural areas, of which the majority are faith-based and other not-for-profit, non-governmental organisations.

### 3.3 Healthcare systems impact on SF medicines and access to quality medicines

Health systems in Africa are still characterised by poor infrastructure. The limited availability and access to advanced diagnostic tools in most public and private healthcare systems in African nations impacts the correct and timely diagnosis of the causative agent in the case of microbial infections. This limitation not only delays appropriate treatment but also make it difficult to distinguish true treatment failure from failure resulting from using SF medicines. As a result, the impact of SF medicines often goes without being detected.

**FIGURE 3:**
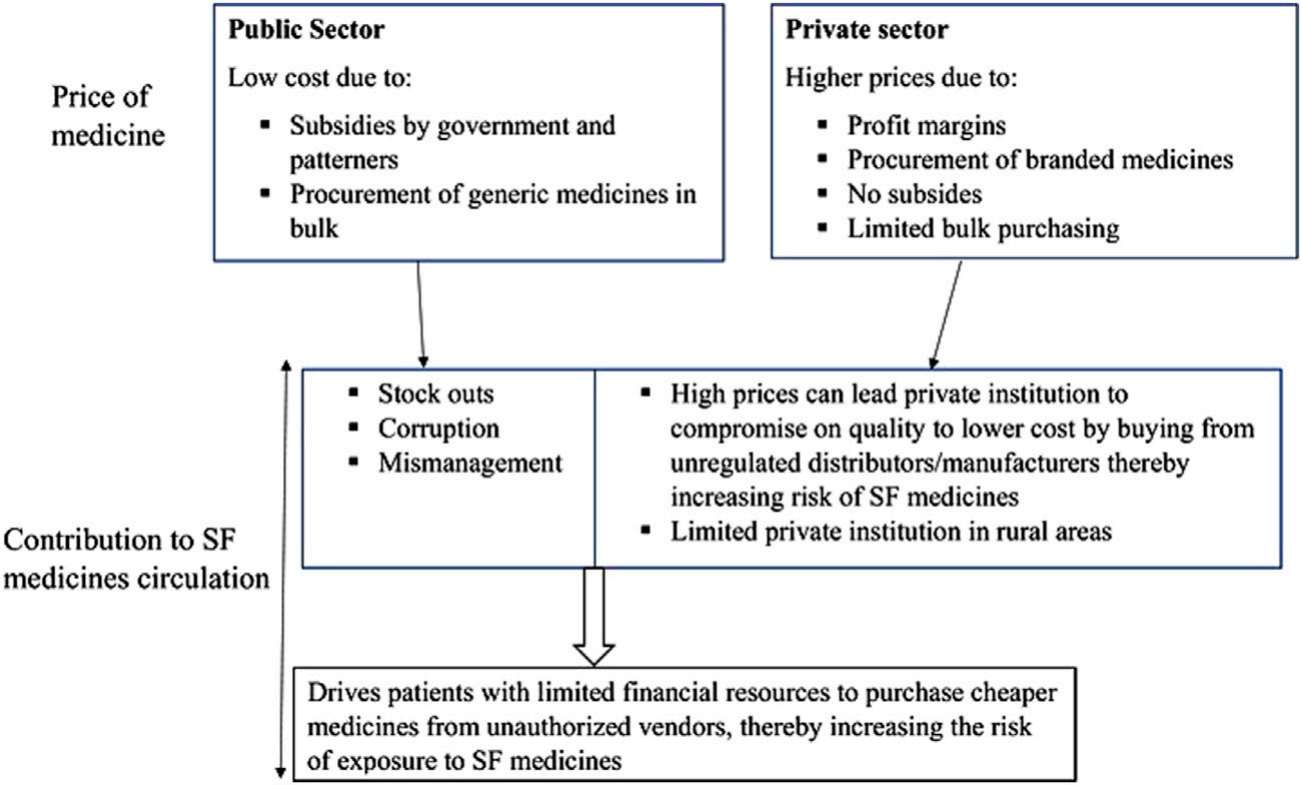
Illustration of how price disparity between the public and private health sectors in Africa plays a significant role in the prevalence of SF medicines.

The cost of treatment between public health facilities and private institutions in Africa is significantly different. For example, in Malawi, the total cost of treatment using some essential medicines was found to surpass the daily earnings of a low-wage government employee (Chiumia et al., 2024). In South Africa, a study carried out on 74 medicines brands revealed an average price differential of 395.47% between the public sector and private sector medicines (Thaver et al., 2021). These disparities in prices are likely to significantly impact the prevalence of SF medicines, as illustrated in Figure 3. While public health institutions often subsidise medicines costs, this is often for a few essential medicines which may run out of stock or are mismanaged. On the other hand, the private sector’s high prices of medicines make them unaffordable to the poor majority in Africa. When patients cannot afford to buy medicines from these private institutions, they are forced to buy from unregulated vendors and mobile street hawkers at cheaper prices, increasing the chances of them consuming SF medicines. A recent study in the northern zone of Cameroon revealed that 87% of individuals bought medicines from unregulated vendors for convenience and social-cultural reasons in addition to cheap medicines compared to regulated pharmacy outlets (Clémence, 2024).

Even with the efforts of decentralising healthcare services, the location of public health facilities in most African countries is traditionally influenced by politics and pragmatic considerations which affects access to quality medicines for the marginalised population. A recent study estimated the geographical access to health institution in sub-Saharan Africa and found out that health facilities in cities and peri-urban areas are easily reachable within 30 min while in rural areas, patients have to travel for more than 3 h to access any health post (Florio et al., 2023). The scarcity of private healthcare systems in most rural parts of African nations as highlighted above under the structure of the private healthcare care system in Africa and long distances to public healthcare facilities is a challenge. Additionally, the fragmentation of some of the public healthcare systems leads to inefficiencies and long waiting time, making patients avoid vising public institutions or forgo follow-ups. As highlighted above this makes consumer seek medical services from informal providers and make the unregulated supply chain to grow. For example, in the rural parts of Uganda, these informal and unregulated traders account for nearly 77% of the healthcare services (Buchner et al., 2019). In most cases, these sell low-cost medicines and often poor-quality medicines.

## 4 Supply chain challenges and impact on the circulation of SF medicines

**FIGURE 4:**
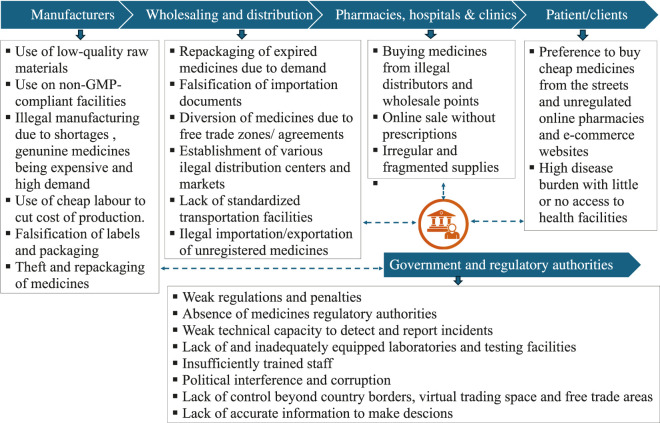
Interconnected factors that drive the higher prevalence of SF medicines across the supply chain.

Availability and access to quality assured medicines depend on the demand and supply systems. A typical supply chain for medicines in Africa is complex as it may involve various manufacturers, a network of distributors in various countries who supply to hospitals and retail outlets. This complexity in the supply chain system creates numerous opportunities to disrupt the supply of quality and safe medicines through unethical and illegal practices across the supply chain levels (Figure 4). The factors contributing to the high incidences of SF medicines across the supply chain are explained below.

### 4.1 High medicine demand and inadequate stock availability

The disease burden on the African continent drives the demand for medicines, with up to 80% of patients depending on public health institutions to access medicines. Unfortunately, medicines in these facilities are not readily available at all times and when available, the stock levels are usually below the 80% threshold recommended by the WHO for medicines availability. For example, a 2019 survey by the World Health Organisation showed that only 36% of African countries reported the availability of essential medicines in the public health sector for non-communicable diseases (World Health Organisation, 2020). Additionally, the 2024 Afrobarometer report revealed that on average, 66% of all Africans visiting public health institutions go without accessing medicines with the percentage rising to 72% for those in rural settings (Lionel and Matthias, 2024). This shortage and low availability of medicines is usually compounded by several factors such as poor forecasting of the supply and demand, diverse regulatory frameworks, weak policy implementation and lack of support to local logisticians (Vledder et al., 2019). In some African countries like Sudan, stockout of essential medicines is also caused by the availability of limited suppliers to meet the demand (Hemmeda et al., 2024). Theft and diversion of the little available medicine in public health institutions occur frequently, also leading to stockout of the quality and safe medicines in public institutions. For example, in Malawi, billions of dollars are lost every year by the government and donors, with the government losing an estimated 30% of the medicines and around 35% of the stolen medicines ending up in private healthcare facilities and black markets (Kateta, 2022). However, detecting diverted or stolen medicines is often difficult due to the sophisticated networks created along the supply chain. Traditional methods such as reviewing monthly or daily medicines usage against issued prescriptions are often inadequate as the records can easily be falsified. As a consequence of medicines shortages and stock outs, a demand is created which then drives the sale and distribution of SF medicines through unethical practices by distributors and suppliers. This also escalates patients’ out-of-pocket expenditure and affects African nations’ ability to make significant progress towards achieving universal health coverage (Hsiao et al., 2019). In some regions, out-of-pocket expenses are high, for example, in East African countries, this constitutes as high as 40% of spending by patients (Steele et al., 2024).

### 4.2 Shortage of qualified personnel at service delivery points

The shortage of trained healthcare professionals in most African healthcare institutions coupled with high levels of untrained staff operating illegal retail outlets increase irrational prescribing and use of medicines (Toroitich et al., 2022). This affects access to medicines and also increase the entry of poor-quality medicines into the Africa healthcare systems. This is as a result of a lack of awareness of the quality and safety of medicines by these untrained individuals who are involved even in the procurement of medicines. For instance, a study in Kenya found that nearly 50% of non-pharmacy-trained personnel were involved in procuring medicines for private health facilities (Abuga et al., 2019). The involvement of unqualified personnel in medicines procurement and lack of standards for supply chain management also is a challenge affecting the private sector in Africa. For example, in Nigeria, 84% of supply chain companies had inadequate standards required for effective management of the medicines supply chain (Chukwu et al., 2018). Additionally, unethical practices by pharmaceutical representatives may influence brand prescribing and dispensing patterns by healthcare providers while pegging prices based on perceived quality. This in turn may force patients to opt for cheaper sources which are often selling poor quality medicines (Toroitich et al., 2022; Abuga et al., 2019).

### 4.3 Inadequate budgeting and complex procurement

While the devolving of public health institutions aims to improve access to essential medicines by having health services closer to communities, it possesses challenges in resource availability. Access to quality and safe medicines in public institutions is impeded by poor financing. In Africa, health finance suffers huge setbacks as the majority of governments fail to allocate 15% of their total national budget for healthcare expenditure as outlined in the 2001 Abuja declarations (Adebisi et al., 2022). The public health sector is left fragmented as a result of limited fiscal space to ensure the availability of quality and safe medicines at all times, in turn causing high stockout levels highlighted above. Healthcare providers in hospitals, including private pharmacies may unknowingly procure and dispense poor-quality medicines due to constrains in budgets. Falsifiers of medicines often may target poor countries in Africa as the retail prices of genuine medicines may be prohibitively expensive for a significant portion of the population creating an alternative cheaper market.

In countries such as Uganda, Sudan and Ghana where the centralised system for the supply of medicines is used, the governments are the main distributors of medicines to public health institutions. Timely access to quality medicines at service delivery points becomes a challenge due to lengthy and complex procurement processes, and unreliable transport systems by government central stores. Medicines procurement is made complex due to uncoordinated timing in budgeting cycles, fund availability, and missing specifications for the medicines to be procured. Additionally, mid-term changes to medicines specifications requirements on awarded contracts, budget cuts and slow initiation of the procurement after tender are awarded makes the process complex (Sorato et al., 2024). This complexity ultimately affects the availability of quality medicines and creates room for substandard medicines infiltrating the supply chain systems due to demand for unmet medical needs as highlighted above.

### 4.4 Increased internet use

With over 570 million people in Africa using the internet as of 2022, the digital market is growing rapidly making people using the internet for health information and buying medicines (Galal, 2024). However, the actual prevalence of people buying medicines online in Africa is not widely documented. The increase in internet accessibility and usage promotes various digital activities including irrational use of medicines as patients can easily self-prescribe and buy medicines online without a prescription in some regions (Jamee et al., 2022). As a result, consumers may easily buy SF medicines from unqualified individuals and illegal online sellers who sell cheap medicines for profit. Criminals are motivated to use online platforms as they easily reach many customers without being traced (World Health Organisation, 2017a). Moreover, they often advertise medicines at very low prices making them very appealing to poor African consumers who cannot afford expensive medicines in regulated private health facilities.

### 4.5 Low production capacity and high production cost

**FIGURE 5:**
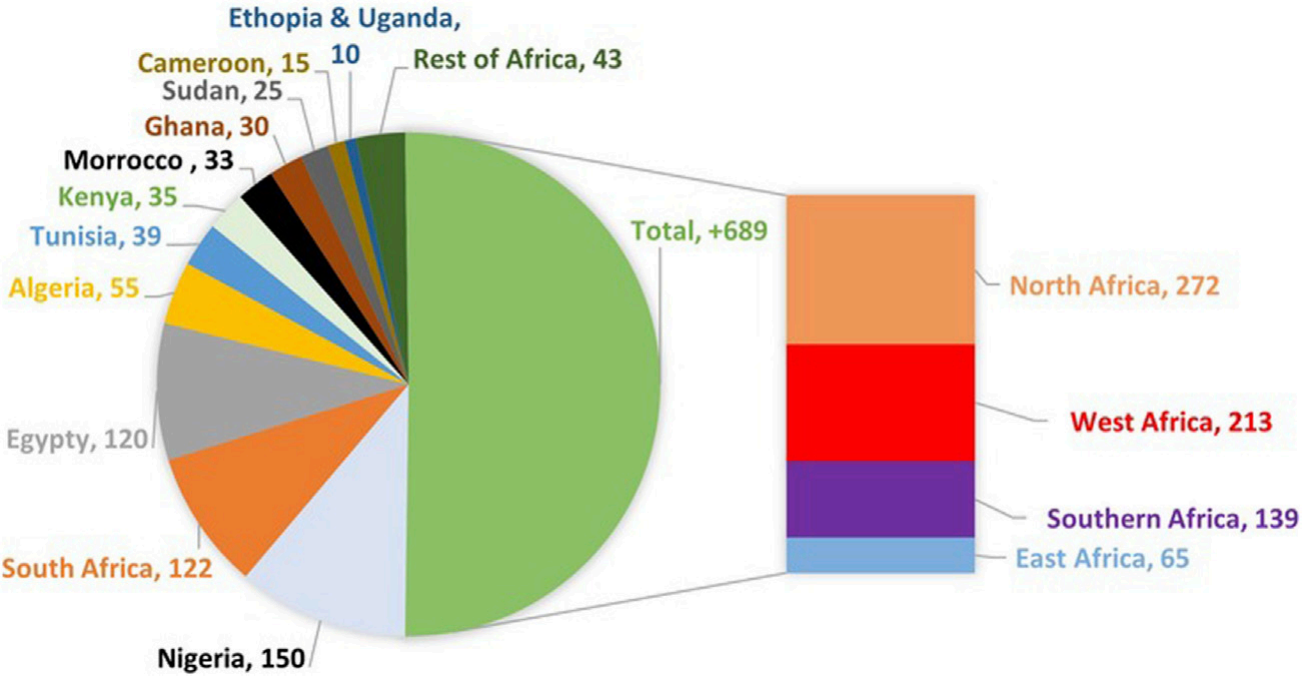
Number of pharmaceutical manufacturing companies in African countries with more than 10 plants (the rest of Africa had less than 10 plants with 22 not having any at all) and regional distribution of the companies (Banda et al., 2022).

Africa is characterised by limited investment in medicine manufacturing, research and development which affects the availability and access to locally manufactured medicines (Ekeigwe, 2019). By 2022, the continent roughly had only about 689 manufacturers (Figure 5) lagging nearly 20 times behind India where there are more than 10,500 manufacturers (Conway et al., 2019; Banda et al., 2022). The available manufacturing capacity remains highly limited and unevenly distributed, with about 80% of the production concentrated in eight countries. Half of these countries are in north Africa, collectively accounting for about 85% of the continent’s roughly 689 manufacturing facilities, while around 27 countries lack any domestic production capacity (Banda et al., 2022; United Nations Conference on Trade and Development, 2025a). For vaccines manufacturing, there were only four countries involved, namely, Egypt, Senegal, South Africa and Tunisia. With respect to manufacturers of active pharmaceutical ingredients (APIs), Africa only has 3 manufacturers, of which two are located in South Africa and one in Ghana (Conway et al., 2019). This limited availability of industries in most African countries encourages the proliferation of illegal manufacturers to fill the gap. These individuals may also falsify the security features of a genuine medicine such as labels, logos, and seals that may be of use for preliminary authentication. Additionally, they may collect and use discarded packaging materials of genuine medicines or relabel expired medicines (Popoola et al., 2022).

These limited available manufacturers on the continent also face challenges such as the high cost of production as they strive to meet stringent regulatory requirements. In Africa, electricity costs is high coupled with excessive power outages in some regions, which deter efficient medicines manufacturing, thus resulting in raised production costs (Adebisi et al., 2022). Such challenges have a potential to make manufacturers compromise on good manufacturing practices requirements as a means of cutting costs that lead to the production of substandard medicines.

### 4.6 High import dependency

The limited availability of finished product manufacturers and API manufacturers highlighted above has led African countries to be overly dependent on importing medicines from other continents. For example, 95% of medicines and 99% of vaccines available and accessed by patients in Africa are imported from outside of Africa (Saied et al., 2022). The heavy dependency on imports also makes African nations struggle to monitor the influx of millions of medicines arriving through various airports, land borders, and seaports from numerous manufacturers. Consequently, they frequently fail to seize all SF medicines and assume their quality is acceptable.

Suppliers of medicines often incur expenses in foreign currency, heavy import taxes and mark-up regulatory fees or charges to ensure medicines are supplied to their markets. In return, medicines become pricier for these suppliers to make profits. For example, in Kenya, it was reported that imported innovator and generic medicines were 32.6 and 2.9 times respectively pricier on average than the median global prices (Ongarora et al., 2019). Such high prices force patients to sources for cheaper medicines from unregulated markets as highlighted above in Figure 3, increasing the likelihood of consuming SF medicines.

### 4.7 Inadequate trade policies

Inadequate trade policies and agreements between countries also contributes to the proliferation of SF medicines. Due to weak trade policies, illegal manufacturers and distributors exploit regulatory gaps, and grey trade connections to facilitate falsified medicine entry using legitimate distribution routes. For instance, illegal manufacturing may be done in one country and packaging in another country, with the final falsified medicine concealed, smuggled or documents falsified to be of another product during export to the final importing African country. The increase in use of courier services in some African nations without proper regulations my pose a challenge in ensuring access to quality medicines, as criminals may use these transport systems to invade regulators. However, the impact courier services on infiltration of SF medicines is not well documented to date in Africa.

The establishment of digital trading spaces has introduced an additional complexity to the supply chain making it a challenge to monitor international markets and combating illegal trade activities. The absence of national territorial borders in virtual space coupled by non-availability or differences in legal frameworks in different African countries makes the detection of falsified medicines difficult. This renders national medicines regulatory authorities (NMRAs) less effective in addressing this major public health issue. Ultimately the quality of medicines circulating in most countries becomes compromised.

Lack of price regulations enforcement and entirely unavailability of such policies for medicines in most African countries affects medicines availability and cost as highlighted above. For example, the prices of medicines distributed by the central medical stores in Sudan were found to be nearly twice the price set by the procuring agency demonstrating a lack of price control (Lucero-Prisno et al., 2020). Additionally, the prices of imported medicines in Sudan were ten times pricier than the global reference prices (Lucero-Prisno et al., 2020). This lack of regulation and control of medicine prices ultimately affects timely access to quality and safe medicine. Trade-related issues such as patents additionally impede medicines availability in most African nation and access by monopolising prices (Tenni et al., 2022). This, in turn, creates room for marketing cheap SF medicines to meet the demand for medicines on the continent (Sorato et al., 2024; Yenet et al., 2023).

### 4.8 Inadequate penalties for medicines falsification

The sale of SF medicines can also thrive in nations when legislation necessary to impose appropriate criminal penalties and punishments for offenders are inadequate. In some African countries, penalties for drug falsifiers are significantly milder compared to those for drug traffickers, who often face substantial fines and lengthy prison terms upon successful prosecution. In Tanzania, for instance, the penalty for dealing in SF medicines was five million shillings (less than US$2,000) and 2 years imprisonment (Karungamye, 2023). Similarly, in Nigeria the penalty was a fine of five hundred thousand Naira (equivalent to US$1,200 as of 2021) and/or imprisonment not exceeding 15 years (Akpobolokemi et al., 2022). The relatively lower penalties compared to the trade in cocaine and other substances make medicines falsification attractive and African countries with weak healthcare regulatory laws and legal systems become targets.

### 4.9 Bad governance

Bad governance, political interference and corruption in most African nations weakens the capacity of medicines regulatory authorities, resulting in insufficient inspection, regulation and supply of medicines without proper licenses. Suppliers may create networks by corrupting individuals in key institutions of governance, which further weakens the capacity to remove poor-quality medicines from legitimate supply chains.

The complex nature of the supply system as demonstrated above and highlighted in Figure 4, although not specific to Africa alone impact tracing the origin and distribution path of each product, and if SF medicines infiltrate the system, it becomes difficulty to trace. The factors highlighted above synergistically contribute to high incidences of SF medicines on the continent.

## 5 Regulatory challenges

Most African countries have either a National Medicines Regulatory Authority (NMRA) or a unit under their Ministry of Health that conducts surveillance of medicines quality in their markets. They also inspect manufacturers and other players along the pharmaceutical supply chain, register medicines, and conduct quality analytical testing of medicines among other functions. However, most African NMRAs are faced with numerous challenges that have a direct impact on their performance in ensuring the availability of quality, safe and efficacious medicines, and protecting public health. These include a lack of well-resourced regulatory systems, inadequate skilled personnel, and inadequate laboratory facilities for conducting routine screening and confirmatory medicine quality tests. In this context, screening is the term for testing and evaluating of medicines including the package to assess if they meet the set standards of quality while confirmatory tests are additional tests conducted to verify that the medicine is indeed of good quality, a substandard or falsified.

### 5.1 Sub-optimal regulatory systems

**FIGURE 6:**
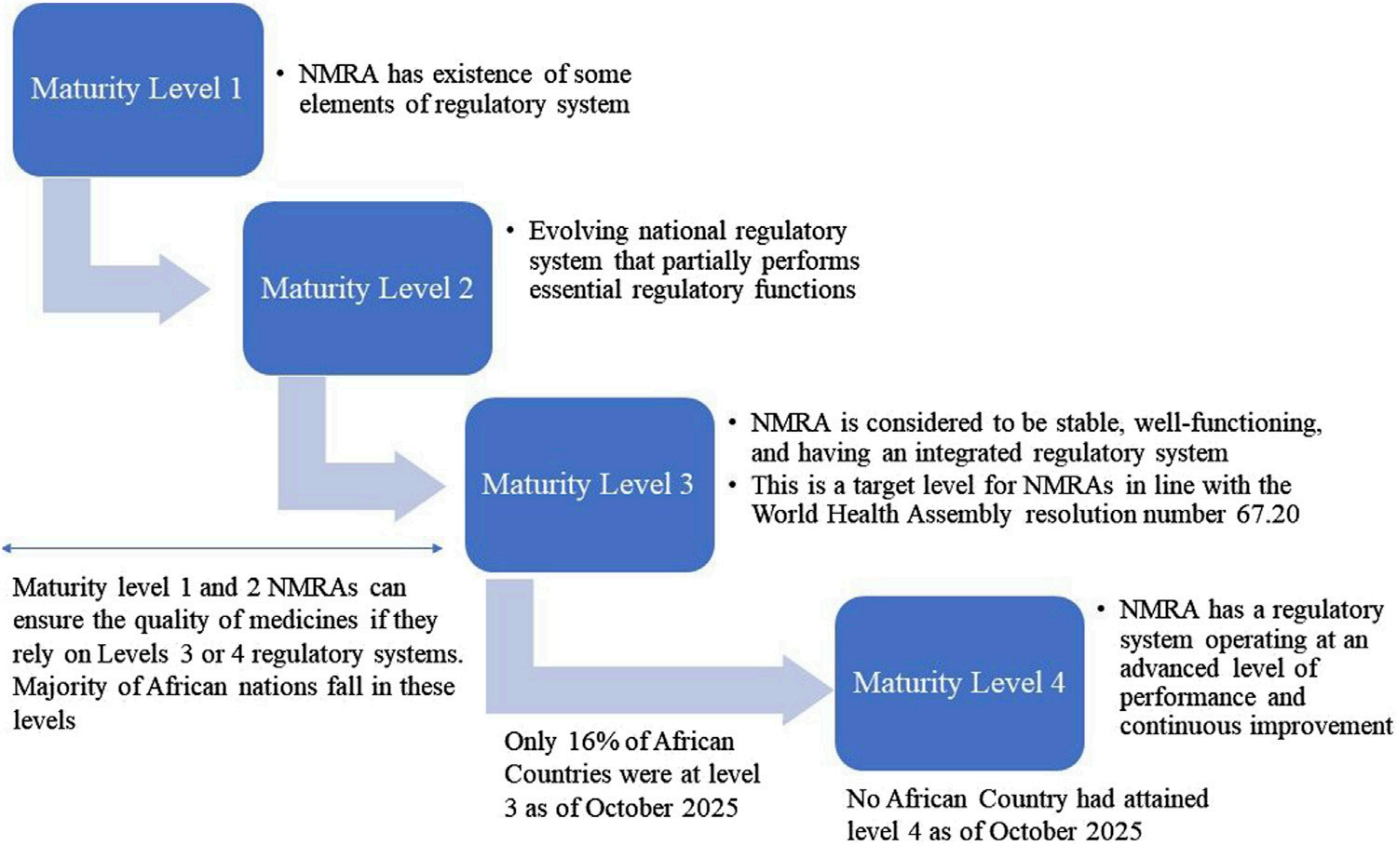
The WHO GBT performance maturity levels for evaluation of national regulatory systems.

A well-established and integrated regulatory system is key to guaranteeing the availability of quality medicines in any country. However, regulatory capacity varies across African countries like in other low resource nations. The WHO developed a Global Benchmarking Tool (WHO GBT) for assessing regulatory capabilities of NMRAs. The WHO GBT assesses NMRA’s ability to perform regulatory functions such as marketing authorisation; licensing; pharmacovigilance; market surveillance and control; clinical trials; inspections; laboratory testing and lot release of vaccines (World Health Organisation, 2021). For evaluation, the WHO GBT uses maturity levels on a scale of 1–4 as presented in Figure 6.

The desirable level for an NMRA to be considered as having a stable, well-functioning and integrated regulatory system able to execute all regulatory functions is maturity level 3 (World Health Organisation, 2021). However, nearly 84% of African NMRAs have weak regulatory systems and are still working to create stable, well-functioning, and integrated regulatory environments. As such, they operate below maturity level 3 with only nine African NMRAs operating at maturity level 3 (Table 2) as of October 2025. The lack of and/or suboptimal operation of key regulatory functions makes these African countries more vulnerable to the proliferation of SF medicines and affect timely access and monitoring of the quality of medicines. As a result, SF medicines can easily make their way into the pharmaceutical supply chains systems and reach consumers.

**TABLE 2:** African countries operating at WHO Global Benchmarking Tool maturity level 3 as of October 2025^a^ (World Health Organisation, 2025).

Country	Name of NMRA	Year reached maturity level 3	Scope of products reached for level 3 performance
Egypt	Egyptian drug authority (EDA)	2022	• Vaccines (producing)
Ethiopia	Ethiopian food and drug authority (EDA)	2025	• Medicines • Vaccine (non-producing)
Ghana	Food and drugs authority (FDA)	2020	• Medicines • Vaccines (non-producing)
Nigeria	National agency for food and drug administration and control (NAFDAC)	2022	• Medicines • Vaccines (non-producing)
Rwanda	Food and drugs authority (Rwanda FDA)	2024	• Medicines • Vaccines (non-producing)
Senegal	Agence sénégalaise de réglementation pharmaceutique	2024	• Medicines • Vaccines (non-producing)
South Africa	South african health products regulatory authority (SAHPRA)	2022	• Vaccines (producing)
United Republic of Tanzania	Tanzania medicines and medical devices authority (TMDA)	2018	• Medicines • Vaccines (non-producing)
Zimbabwe	Medicines control authority of Zimbabwe (MCAZ)	2024	• Medicines • Vaccines (non-producing)

^a^ The list was verified on 25th October 2025. As of this date, no other country was added to the list.

**FIGURE 7:**
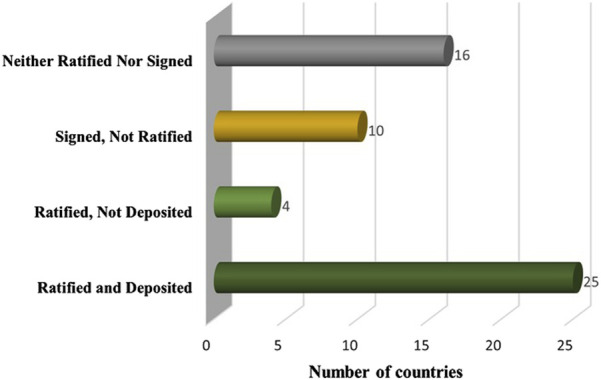
Ratification and signing status of the African Medicines Agency Treaty by as of March 2025.

In an effort to harmonize and strengthen regulatory systems in Africa that ensure medicines circulating on the continent meet the standard of quality, safety and efficacy, the African Union model law on the regulation of medical products was adopted in 2016 (Dube-Mwedzi et al., 2020). African countries are required to ratify and deposit the instrument to affirm their alignment with the African Medicines Agency treaty. Unfortunately, only 25 out of 55 African countries (Figure 7) had both signed and deposited the instrument as of March 2025. Some challenges faced by African countries yet to ratify the treaty include lack of political commitment by parliaments to revise weak legal and regulatory systems to align with the model law, varying interpretations of the benefits and risk of the model law, and insufficient financial mechanisms (Ncube et al., 2023).

### 5.2 Limited funding for medicine quality surveillance

Another regulatory challenge facing most African NMRAs is that they have lean budgets to execute most of their regulatory functions leading to poor regulation of both the private and public markets. The lack of adequate funding hinders some NMRA in Africa from conducting thorough and randomized drug sampling to ensure quality of the available medicines. As such, NMRAs conduct routine surveillance only when resources are available and not all medicines undergo routine surveillance. In most cases, NMRAs rely on reports made by health personnel and patients through their pharmacovigilance reporting systems. This limitation allows SF medicines to proliferate freely, especially in informal markets where regulatory oversight is minimal as highlighted above under supply chain challenges. Additionally, most pharmacovigilance systems and post marketing surveillance systems in African countries are non-functional and characterized by low reporting on medicine quality issues. For instance, a 2020 study analysing regulatory systems in 16 Southern African countries revealed varying levels of pharmacovigilance frameworks and governance systems, which also were characterized by low reporting rates (Dube-Mwedzi et al., 2020). A similar observation was made in another study that assessed the national pharmacovigilance systems in four East African countries (Barry et al., 2020). Pharmacovigilance is a crucial tool in monitoring medicines quality and safety as the reported adverse drug reactions, irrational use of medicines, medication errors and overdose can be a first indication of SF medicines and prompt quick regulatory action (Pozsgai et al., 2022). For instance, in East African nations, 30 signals reported from Kenya and 5 from the United Republic of Tanzania were associated with SF medicines (Barry et al., 2020). In many African countries, data mining from pharmacovigilance databases is very rare, yet routine data analysis of these databases can provide potential clues, patterns and trends to identify SF medicines and possibly reduce the clinical and economic cost of SF medicines (Magro et al., 2020; Toroitich et al., 2024). However, the low reporting levels and inconsistencies in the data reported may hinder the correct mining of pharmacovigilance databases for potential signals of SF medicines.

### 5.3 Limited technical qualified and skilled regulatory personnel

NMRAs in Africa also struggle with staffing levels due to a scarcity of qualified and skilled personnel, high turnover migration for better opportunities, and insufficient continuous education (Adebisi et al., 2022). This tends to affect most regulatory functions, for example, delays in processing marketing authorisation which in turn lead to shortages of quality assured medicines and ultimately hampers the efforts by African governments to achieve universal health coverage. Marketing authorisation timelines in Africa are still unpredictable with typical lag times of up to 7 years from the time of application (Ekeigwe, 2019). These lengthy registration periods affect access to quality-assured medicines as they are among the reasons manufacturers and suppliers tend to be reluctant in supplying medicines as their market planning is affected. The human resource challenge also affects import inspections at ports of entry as there are no dedicated staff to carry out this function and, in most cases, no proper surveillance of both the private and public markets as quality assurance efforts are limited to public procurement (Preston et al., 2020). Additionally, there may not be staff dedicated to handling reports of adverse events and SF medicines in some African NMRAs.

### 5.4 Limited analytical technologies and prequalified laboratories

Testing of medicines is crucial for ensuring the quality, safety and efficacy of medicines to protect public health by supporting regulatory decisions. This is done through screening and confirmatory testing of medicines on the market as stated above. The main analytical techniques used for the screening and confirmatory tests during the surveillance of medicine quality in most African countries are summarised in Table 3.

**TABLE 3:** Summary of analytical techniques and tools used by most African NMRAs for surveillance of medicines quality.

Application	Technology/technique/tool	Role
Visual examination, weight and physical dimensions of sample	Check list and balance	Screening
Formulation performance	Disintegration Dissolution	Screening confirmation
Formulation analysis; API, impurities, residual solvents identification and quantification	Colorimetry	Screening
	Separation technique, e.g., thin layer chromatography	Screening^a^ Screening^b^ Confirmation
	Spectroscopic technique	
	Separation technique, e.g., high performance liquid chromatography, gas chromatography	

^a^ Can be used on a limited basis for quantification of API and impurities.

^b^ Limited evidence exists in the literature on African NMRAs, using the technique.

However, many laboratories in Africa are underequipped and remain far from meeting global standards. For example, of the 10 WHO-prequalified laboratories on the African continent (Table 4), only 6 are operated by 6 NMRAs in Ghana, Kenya, Nigeria, Tanzania, Uganda and Zimbabwe (World Health Organisation, 2024). As a results, the majority of NMRAs in Africa conduct laboratory analysis in non-WHO-prequalified sites facing several analytical challenges that have an impact on public health. Firstly, the majority rely on pharmacopoeial methods that typically require a large sample size of over 20 units to carry out analysis such as assay and dissolution. Such sample size may not be available making it not feasible to conduct several analytical tests. As such, medicines may be considered to be of good quality without carrying out all critical test parameters. Moreover, extensive sample preparation is required to undertake such tests which impact the speed of analysis and often requires skilled personnel to carry out such sample preparations.

**TABLE 4:** List of prequalified quality control laboratories in Africa as of June 2024 (World Health Organisation, 2024).

Name of organization	Country	Operated by NMRA	Year of prequalification
Research institute for industrial pharmacy (RIIP) incorporating CENQAM	South Africa	No	2005
Adcock ingram ltd research and development	South Africa	No	2008
National quality control laboratory (NQCL)	Kenya	Yes	2008
Mission for essential drugs and supplies (MEDS)	Kenya	No	2009
Tanzania medicines and medical devices authority (TMDA)	Tanzania	Yes	2011
Medicines control authority of Zimbabwe (MCAZ)	Zimbabwe	Yes	2014
National drug authority of Uganda	Uganda	Yes	2015
M And L laboratory services (pty) ltd	South Africa	No	2017
Food and drugs authority (FDA)	Ghana	Yes	2022
National agency for food and drug administration and control	Nigeria	Yes	2023

As a result of high volumes of medicines required for the high disease prevalence on the continent, many laboratories do not have the capacity to test the high volumes of products and cannot fully implement monograph testing resulting in many medicines not being tested particularly those from smaller or well-known manufacturers. Additionally, non-pharmacopoeial protocols also called in-house methods are not readily available for use by NMRAs in Africa. This has a potential to increase the number of SF medicines reaching the patients due to lack of testing. Accessing high-purity reference standards, solvents, reagents, consumables and compressed gases required for routine operation is also a challenge in using confirmatory analytical techniques in Table 3 (Bakker-’t Hart et al., 2021). Therefore, in most cases, conducting confirmatory tests may take several days which impacts regulatory decision time. Additionally, high maintenance cost, frequent power outages, high temperatures, and humidity, as well as limited after-sale support for the analytical instrument make it difficult to operate modern instruments on a routine basis. Furthermore, spare parts for equipment maintenance have to be imported and delivery usually takes days to several months. Moreover, most laboratories often do not have adequate funds to enable procurement of advanced analytical equipment as falsification of medicines becomes sophisticated. In some instances, they end up using outdated equipment or malfunctioning ones reducing the accuracy and reliability of the results. Finally, most African countries are faced with geographical barriers for quality laboratory testing. The quality control laboratories are mostly concentrated in urban area, making it difficult to conduct confirmatory tests in other regions and borders of the country without sending samples to the national quality control laboratories. This also causes logistical problems for the transportation and storage of samples.

## 6 Strategies to increase access to quality medicines and reduce incidences of SF medicines

While significant progress has been made in increasing access to quality medicines in Africa, several gaps need to be addressed to reduce the prevalence of SF medicines and move closer towards universal access to quality medicines for all. Ensuring that quality, safe and effective medicines circulate in African countries requires involving all key players across the supply chain of medicines from manufacturers to the consumer. The various strategies required at all levels to enhance access to quality medicines in Africa and reducing the circulation of SF medicines are discussed below.

### 6.1 Implementation of international agreements

A number of commitments have been made by governments in an effort to improve the healthcare system in Africa. One such example is the 2001 Abuja declarations, however, over the past 24 years, most African governments have struggle to allocate 15% of their national budgets to healthcare. Implementing these agreements is key to healthcare financing which ultimately will improve access to quality medicines more particular in public healthcare institutions where the majority of the African population access medicines. Governments should ensure that policy inconsistencies are dealt with by streamlining them to declarations and commitments made in order to achieve universal health coverage.

### 6.2 Rational use of medicines

Policies for rational use of medicines should be strengthened, and efforts should be made to ensure all service delivery points adhere to these policies. Deliberate policies should be considered to ensure both the public and private healthcare sectors adhere to standard treatment guidelines that promoted rational use of medicines. This will ensure that correct medicines are dispensed for the correct diagnosis and thereby reduce wastage or overconsumption due to empirical treatment. Ultimately, this has a potential to reduce the demand for cheaper SF medicines when medicines are available in public health facilities and used correctly.

Developing policies that promotes prescribing, and procurement of generic medicines is essential. For example, the implementation of such polices in seven African nations reduced the cost of antiretroviral medicines by 40% compared to the cost of buying innovator products (Koduah et al., 2022).

### 6.3 Human resource development

Human resource is still a big challenge at all levels in Africa despite efforts made to increase staffing levels. At health facility level, ensuring the right cadres are employed and providing on-going training, sensitisation and continuous professional development to all healthcare providers in identifying SF medicines should be emphasized. Additional strategies should include the provision of education grants to healthcare professionals to undertake advance training.

For pharmacy students in Africa, incorporating training on SF medicines into pharmacy degree curriculums such as modules developed by the International Pharmaceutical Federation should be considered across African universities. Pharmacists are gatekeepers in medicine management, and it is crucial that they are aware and well trained on identifying and handling SF medicines to protect public health. As of 2024, some universities in Cameroon, Senegal, Tanzania and Uganda had implemented this curriculum into their training (Kusynová et al., 2023). Implementation of this curriculum showed that the knowledge of pharmacy students on SF medicines in these Africa countries increased by 3.5 score points (Kusynová et al., 2023).

As with the shortages of healthcare professional in health institutions, Africa pharmaceutical industry struggle with a shortage of industrial pharmacists and laboratory staff with the relevant experience and capability attributed to insufficient academic pharmacy education. Some manufacturers resort to using other professionals to bridge the gap in skills. For example, in Tanzania, a study found that some production staff were nurses employed due to their basic understanding of the importance of hygiene (Banda et al., 2022). Therefore, in house skills transfer is key for staff development. Industries should also work with universities to create tailored trainings for continuous professional development for industrial pharmacist. Additionally, universities in Africa should align undergraduate pharmacy curriculums to the needs of the manufacturing industry. The right trained industrial staff are key to ensure quality assured medicines are produced and supplied on the continent.

Development of competent regulatory workforce in all key functions of NMRAs is another action that can enhance the efforts to prevent SF medicines incidents. Access to safe quality and effective medicines can never be realized if there are limited competent regulators in all regulatory functions. Currently, there has been much emphasis on training medicines dossier assessors, GMP inspectors, pharmacovigilance experts and laboratory analysts through regional collaboration and centre of excellencies. This need to be extended to officers involved in post-market surveillance of medicines as a key component in the fight against SF medicines. Training should focus on identifications of SF medicines, how to use simple and rapid field deployable analytical instruments, development of screening protocols and strategies for sampling medicines. In addition to the current trainings conducted by various regional centre of regulatory excellence, there is a need of expanding access to regulatory fellowships, internship and exchange programs. This should be done as a strategy to develop skilled personnel, motivate them and ensure there is continuous skills development. Additionally, improvement in renumerations for regulators to prevent staff leaving for better paid jobs is crucial.

### 6.4 Enhanced consumer education

Consumers and patients also play a key role in fighting SF medicines by reporting incidents of SF medicines. Therefore, it should be a norm for Africa governments and NMRAs to ensure there are efforts made to conduct public education and awareness campaigns on the impact of SF medicines, importance of reporting incidents and mechanisms available for prompt reporting of incidents encountered by consumers. Being aware of the consequences of consuming SF medicines should be the first step in preventing consumers from buying SF medicines. This is because in some instances, the cause of death may go unnoticed or may not be attributed to consumption of SF medicines. Examples of awareness could be information dissemination through workshops, television advertisements, signs, posters, and school education. Consumer versions of verification checklists should be developed by medicines regulators and ensure they are disseminated through various media including social media platforms. Additionally, strategies to empower consumers to verify authenticity of the medicines such as use of mobile phones should be put in place in all countries as exemplified in Nigeria. The Nigerian National Agency for Food and Drug Administration and Control (NAFDAC) adopted five mobile authentication service schemes (MAS) and developed guidelines for procuring/implementing these schemes by suppliers of antibiotics and antimalarial medicines. The MAS is one of the strategies employed by NAFDAC to combat SF medicines by empowering consumers to verify the authenticity of medicines at the point of purchase through a scratch code and short message service (Nigeria National Agency for Food and Drug Administration and Control, 2018). As the use of mobile phones is now increasing in Africa, this has a potential to protect consumers from taking SF medicines.

### 6.5 Infrastructure development

Health systems strengthening through infrastructure development and upgrading of healthcare systems to improve diagnosis and identification of treatment failure and adverse reactions caused by SF medicines should be an agenda of all African governments. Additionally, technological advancements in digital health have the potential to improve access to medicines, as it can enhance management and distribution of medicines. Deliberate policies should be formulated to encourage the adoption of digital health particularly in far to reach areas where most patients are marginalised and have limited access to medicines. Governments should invest in infrastructure to safeguard medicines from theft and diversion. In addition to physical security to prevent unauthorised access, investing in digital surveillance tools such as cameras and installing electronic logistics information management systems that require use of biometric login should be encouraged to avoid data falsification. This will minimise the risk as the focus shifts from individual accountability to system-wide strategies. Moreover, accurate forecasting of consumption data can be achieved to avoid stockouts of quality essential medicines when technology such as artificial intelligence is used correctly, while allowing the limited available healthcare workforce perform all their functions rather than manual inventory systems. Investment in infrastructure for proper storage of medicines at central stores, regional stores and service delivery points should be committed to by governments in Africa and implemented. This will ensure medicines maintains their shelf life before patients can access them.

Preventing SF medicines from entering the supply chain is ideal, but it is also crucial to detect them throughout the supply chain system. Strategies employed currently include border control, implementing a proper reporting system, employment of risk-based inspections and surveillance, and using physical and chemical laboratory analytical techniques to confirm SF medicines (World Health Organisation, 2017a). However, with only 6 African NMRAs having their quality control laboratories reaching the WHO prequalification level (Tale 3), this calls for increased investment and facilitating easy access to analytical technologies by most African regulators. Firstly, the number and capacity of laboratory operating at the WHO prequalification level need to be increased. Secondary, investment in research and development of faster analytical techniques requiring no or less expensive reagents and solvents should be done, and such tools should be adopted for screening of large volumes of medicines on the continent (Tanna et al., 2023). Thirdly, NMRAs currently without capacity or access to detection tools should collaborate with others with capacity within the region, continent or internationally to ensure reliability of the results.

Lastly, enhancing the surveillance system and feedback mechanism is required. Increasing the surveillance of SF medicines should not be limited to actively sampling of medicines, testing and recalling of confirmed cases. Technology advancement should be leveraged for data mining especially pharmacovigilance database, as analysis of pharmacovigilance reports can provide signals to the presence of SF medicines on the market. Moreover, data mining of these database can be a source of evidence required to tighten regulations for high-risk medicines in Africa (Toroitich et al., 2024). However, a barrier to using these databases in Africa is low reporting level. One cause of this, is lack of feedback given to reporters by NMRAs when incidents of side effects, medication errors and medicines quality problems are reported. As such, an individual may feel reluctant to report or feel their efforts will not be recognised. It is therefore imperative that regulators develop feedback mechanisms that values contributions made by reporters with a goal of creating a reporting culture. Such mechanisms could include recognition and certificates of award for reporting, automated emails to reporters and financial incentives, where possible.

### 6.6 Financing and support to local manufacturers

Local production of pharmaceuticals in Africa has the potential to reduce the cost of medicines and increase access to quality medicines as foreign currency demand for imported medicines is reduced. Additionally, supply chain challenges such as shipping delays, global supply chain disruptions, transport cost and delays in clearance at port of entry can be mitigated. One study reported that medicines such as tablets manufactured in Ethiopia and Nigeria could be as cheaper as 15% compared to imported ones from India, however manufacturing capabilities are still a challenge (Conway et al., 2019; United Nations Conference on Trade and Development, 2025a). Majority of African manufacturers operates between 30%–60% compared to well resourced economies in which manufacturers operates at over 70% capacity (United Nations Conference on Trade and Development, 2025a; United Nations Conference on Trade and Development, 2025b). This potential requires more investment in local production of medicines. Access to finances is a challenge for most African medicine manufacturers either at developmental stage or commercial operation. Different financial models should be explored by manufacturers for long-and medium-term funding such as bonds, equity, grants, joint ventures, and syndicated term lending. For short to medium term funding, overdrafts, short term loans, guarantees and in-kind finance are options manufacturers should consider to finance their operations (Banda et al., 2022). Such funds should be invested in improving quality systems of manufacturers within Africa. When the capacity to locally manufacture medicines grows, the market share can potentially grow outside Africa as other global players may procure from such sites to support them in addition to meeting the local demand for medicines. An example is the Quality Chemical Industries Limited in Uganda which has received WHO-prequalification for its antimalarial and antiretrovirals following investments made through a joint venture with several partners. This has enabled the company to supply medicines to over 30 African countries and international agencies such as the Global Fund are able to procure medicines for their programs (Banda et al., 2022). Such funding models enable African manufacturers to have resources required to implement stringent regulation for complying with good manufacturing practices and produce quality medicines.

The local industry in North Africa has actively leveraged joint ventures to facilitate technology transfer. Other regions such as the Eastern and Southern part of Africa where industries are still scares (Figure 5) should leverage on such ventures with the support of government policies to enhance productivity and grow the sectors. Governments within regions of Africa can collaborate and support establishment of technology incubation centres and development of capabilities to locally manufacture APIs for essential medicines. This support has the potential of reducing the cost of production as the cost of importation from the rest of the world is curtailed and consequently reduce patient out-of-pocket expenditures through reduced medicines cost.

Due to the high disease burden in Africa that does not affect the rest of the world, investment in research and development of medicines for neglected diseases is vital to ensure access to readily available medicines. This is because investment in research and development for such medicines may not be economically sound for foreign manufacturers to venture in. Therefore, policies that facilitates research and development using locally produced raw materials and herbal medicines should also be supported.

Governments should provide an enabling environment by creating policies and agreements that deliberately promotes locally produced medicines within Africa. For example, policies that enable allocation of free land leases for industrial parks, subsidies and reduced taxation of imported raw materials and manufacturing equipment. Additionally, governments should increase investment in infrastructure for power supply and transport networks and further provide incentives for energy cost to the industry. Uninterrupted power supply is essential for ensuring medicines are produced timely while good road network ensures smooth transportation to service delivery points. To increase stock availability, efforts should be made to reducing rigidities in the procurement system of quality assured medicines. An example is ensuring that African local manufactures are given long-term tenders for up to 5 years. Timely payment for the medicines supplied to government to ensure manufacturers and distributors have access to revenue for continuous production and supply of medicines is recommended.

### 6.7 Enhanced regulation against SF medicines

Regulatory system strengthening in Africa is one key factor in ensuring that patients have access to quality medicines. However, most African nations have weak regulations to prosecute criminals involved in the supply of SF medicines. This is compounded by poor political will and competing law enforcement priories making trials to take years with very low punishment given to offenders. African nations must therefore ensure that medicines regulation and laws are aligned to the African model law on regulation of medical products, incorporating stiffer penalties and sanctions to offenders commensurate to the crime committed along the supply chain. Another issue that should be addressed is the prevention of corruption through implementation of stiffer regulations. Corruption undermines efforts to strengthen access to quality medicines as it remains a pervasive issue with most African nations.

Many African nation still have no regulations for control the sale of medicines on the internet. Such countries should quickly develop legislation needed to protect public health and ensure that laws, guidelines and policies evolve from time to time with the emerging of newer forms of commerce to ensure SF medicines do not infiltrate through these new channels. Interventions should not only end at putting regulations in place but extend to monitoring websites trading in medicines online across Africa.

Regulations that protect the whole supply chain of medicines is crucial. African governments should ensure policies and regulations related to anti-counterfeiting technologies are in place and aligned with global requirements such as the African model law on regulation of medical product which include legal provision on combating SF medical products as highlighted above. These technologies should digital solutions such as block chain, image processing RFID, mobile phones, 2D data matrix, cryptography and near field communications, serialisation, track and trace, website and dark web monitoring.

### 6.8 Enhance regulatory collaboration

To enhance timely access to quality medicines, harmonisation of regulatory practices in regions and at continental level through the African Medicines Agency must be fast tracked. The current efforts made by countries signing the treaties is commendable, however, creation of legally binding regional frameworks is also key for collaboration. This will ensure consistency in standards across Africa thereby fast-tracking regulatory decisions and potentially attracting more investment in local manufacturing of medicines as duplication of efforts is reduced. Further, NMRAs in Africa should expand the implementation of regulatory systems such as work sharing, reliance or mutual recognition to accelerates licensing and easy access to quality medicines for all. Examples of such initiatives is the recent agreement between the NMRAs of South Africa and Egypt to collaborate in key regulatory functions for pharmaceuticals, biological products and medical devices (South Africa Health Products Regulatory Authority, 2023). There are also other opportunities at a global scale for collaboration that should be explored and adopted as they are less used by some NMRAs such as the ‘EU-M4all procedure’ and the WHO collaborative registration procedures.

Combating SF medicines should be a collaborative effort by all stakeholders. Alerts to other stakeholders is important to prevent further harm posed by SF medical products and infiltration of such products in other regions or even triggering investigations. An example is the case of four contaminated paediatric cough syrups detected in the Gambia in 2022, which led to the WHO issuing an alert, which in turn, probed investigations leading to the detection of the same substandard products in other countries (Thiagarajan, 2022). As such, regulators should ensure that evidence-based policies and procedures for collaboration are available.

### 6.9 Price control strategies

The involvement of several dealers in medicines supply in African nations creates a problem of controlling the prices of medicines. Countries without price regulations should develop regulations and put in place agencies that control prices of medicines. Implementation of different pricing control measures should further be considered. Such measures include enforcing tiered pricing models based on regions or target markets, subsidies and removing regulatory mark-ups by merging various regulatory fees e.g., import licenses and taxes. Price regulation may, however, not always increase access to quality medicines, for example the ever-fluctuating foreign currencies and increase in inflation may affect global prices and finances available to meet the demand. It is therefore imperative that African countries implement sustainable policies, monitor and evaluate these polices to apply country specific mechanisms aimed at ensuring medicines quality is not compromised. Several stakeholders from both public and private sector should participate in designing pricing model as a measure of transparency. The private-not-for-profit organisations in Africa play a key role in medicines access through procurement and distribution of medicines to far-to-reach rural areas, therefore, their voices are crucial in policy development. Stakeholders in academia should also participate in research to ensure such polices are evidence-based.

While it is acknowledged that considerable efforts are being made to ensure quality medicines are circulating in Africa, it is highly recommended that priority should be given in areas that need urgent attention as detailed in Table 5. These priority levels and cost setting are provided to reflect a balance between urgency, feasibility and resource intensity in combating SF medicines in Africa. Interventions such as awareness campaigns, human resource development, and regulatory enforcement can be implemented quickly with the existing resources. Infrastructure improvement, digitalisation and regional manufacturing hubs are assigned longer-term as they are complex and require more finance to implement but remain critical. By balancing the immediate interventions and long-term capacity building across the healthcare systems, supply chain systems and regulatory systems, the framework ensure SF medicines incidences in Africa is collaboratively addressed.

**TABLE 5:** Proposed priority levels for taking action to increase access to quality medicines and reduce the prevalence of SF medicines in Africa.

Category	Summary of recommendation	Cost required	Priority level	Timeframe	Lead stakeholder
Healthcare systems	Implement SF medicines modules in all university pharmacy degree curriculums	Low	High	Medium- term	Universities/ministry of education
	Enhance consumer awareness of SF medicines	Low	High	Short-term	NMRAs, ministry of health (MoH), NGO and media
	Conducting CPD training to healthcare professionals	Low	High	Short-term	Regulators/health professional bodies
	Standardised treatment protocols and rational use of medicines	Medium	High	Medium- term	Ministry of health/healthcare facilities
Supply chain	Use of digital tools, serialisation and track and trace	Medium	High	Medium- term	Ministry of health, NMRAs and manufacturers
	Policies to support incentivise to local manufactures	Low	Medium	Medium- term	Governments through MoH
	Investment in R&D of medicines for africa neglected diseases	High	Medium	Long-term	Governments, donors and manufacturers
	Creation of regional manufacturing hubs	High	High	Long-term	Governments and regional economic communities (RECs)
	Digitalisation and streamlining of procurement process	Medium	High	Medium- term	MoH and procurement agencies
	Digitalised inventory systems	Medium	Medium	Medium- term	MoH and supply chain agencies
	Improving infrastructure for storage and transport of medicines to hospitals	High	High	Long-term	MoH and logistic partners
	Price regulation of medicines	Low	Medium	Medium- term	Governments
	Increased budgetary allocation required for procurement	High	Medium	Long-term	Governments/donors
	Public-private partnership to strengthen supply chain transparency	Medium	High	Medium- term	Governments and private sector players
Regulatory	Introduce stringent penalties for medicine falsifiers	Low	High	Short-term	Governments/NMRAs
	Increased online surveillance of medicine	Medium	High	Medium- term	NMRAs
	Adopting and utilisation of analytical tools and surveillance technologies for the rapid screening detection of SF medicines	Medium	High	Medium- term	NMRAs and quality control laboratories
	Attain at least WHO GBT maturity level 3	High	High	Long-term	NMRAs
	Expand training programs for regulators	Medium	High	Medium- term	NMRA/universities
	Data mining of pharmacovigilance databases for regulatory decisions	Medium	High	Medium- term	NMRA/universities
	Community level reporting and feedback system enhancement	Low	High	Short-term	MoH/NMRAs and NGOs
	Regional collaboration for harmonised regulatory frameworks	Medium	Medium	Long-term	NMRAs and RECs

## 7 Conclusion

Ensuring quality and safe medicines are in circulation is still a major challenge that African governments and regulators have to address. This review has shown how healthcare systems, supply chain and regulatory challenges in Africa impact the high prevalence of SF medicines, thereby limiting access to quality medicines and impacting public health. To reduce the increase in SF medicines circulating on the continent, efforts are needed to always ensure the availability of quality medicines through increased investment in the manufacturing of medicines, adequate funding and rational use of the available medicines. There should also be strong political will, strong regulatory and health systems, adequately trained and skilled personnel, and heightened surveillance. Additionally, fast, reliable and inexpensive testing systems and utilisation of field-deployable analytical technologies, and inspections are required to fight the high prevalence of SF medicines in Africa. The escalating threats of SF medicines in Africa necessitates a multifaceted, collaborative and proactive approach that includes healthcare system strengthening, advocate for policy reforms, prioritising resources to secure the pharmaceutical supply chain and exploring innovations.
